# Protective Effect of Electroacupuncture at Zusanli on Myocardial Injury in Septic Rats

**DOI:** 10.1155/2018/6509650

**Published:** 2018-10-08

**Authors:** Lijian Zhang, Zhenjun Huang, Xian Shi, Sen Hu, Daniela Litscher, Lu Wang, Gerhard Litscher

**Affiliations:** ^1^Department of Rehabilitation of the 309^th^ Hospital of People's Liberation Army, Beijing 100091, China; ^2^Department of Acupuncture and Moxibustion, People's Liberation Army General Hospital, Beijing 100853, China; ^3^Laboratory of Shock and Organ Dysfunction, Burns Institute, First Affiliated Hospital of PLA General Hospital, Beijing 100037, China; ^4^Research Unit for Complementary and Integrative Laser Medicine, Research Unit of Biomedical Engineering in Anesthesia and Intensive Care Medicine, and TCM Research Center Graz, Medical University of Graz, 8036 Graz, Austria

## Abstract

The protective effect of electroacupuncture (EA) at Zusanli points (ST36) on myocardial injury in a model of sepsis was observed. Forty-eight male Sprague-Daley rats were subjected to sepsis by cecal ligation and puncture (CLP) and randomly divided into 4 groups (n=12; A: sepsis + EA; B: sepsis + sham acupuncture; C: sepsis + vagotomy; D: sepsis + vagotomy + EA). Bilateral points were stimulated (2mA, 2-100 Hz) for 1 hour. Abdominal vagotomy was performed in groups C and D. At 6h after CLP, the plasma activity of creatine kinase-MB (CK-MB) was determined. A part of cardiac muscle was harvested for evaluating levels of tumor necrosis factor (TNF-*α*), nitric oxide (NO), myeloperoxidase (MPO), and the rate of water content. The activities of CK-MB, TNF-*α*, NO, and MPO and the rate of water content in group A were significantly lower than those of the other groups 6h after CLP. EA after vagotomy showed less anti-inflammatory and protective effects. The results indicated that EA obviously reduced the increased levels of the proinflammatory factors at 6h after CLP, and vagotomy could weaken or eliminate the effects of EA. Cholinergic anti-inflammatory pathway is one of the main mechanisms of cardioprotective effect of EA.

## 1. Introduction

Serious infection, caused by trauma and major surgery, can lead to sepsis, septic shock, and multiple organ dysfunction syndromes (MODS) [1]. The heart is vulnerable in the process of sepsis, especially ischemia-reperfusion injury caused by tissue ischemia or inflammatory overstimulating [2]. Therefore, suppressing proinflammation and reducing tissue edema play important roles in the prevention and treatment of sepsis.

Acupuncture therapy has been used for thousand years in China, and two-way regulation of nerve-endocrine-immune system. Zusanli point (ST36) can promote the intestinal function and improve immunity [3]. In addition, the modern research has shown that acupuncture at Zusanli can protect organ function effectively from endotoxemia or postoperative abdominal adhesion in rats by inhibiting inflammation factor levels [4–7].

The objective of this paper was to observe effects of electroacupuncture (EA) at Zusanli on myocardial injury in septic rats and to study the anti-inflammatory mechanism of Zusanli.

## 2. Materials and Methods

### 2.1. Experimental Animals and Grouping

Forty-eight male Sprague-Daley (SD) rats, weighing 220 ± 20 g, were obtained from the Animal Center of Military Medical Sciences [Certificate number: SXCL-(Army -2010-006)]. The animals were housed at a constant temperature of 24 ± 2°C and humidity of 50 ± 5 % for one week. They received no food in the 12 hours before the experiment, but had free access to water.

The SD rats were randomly divided into 4 groups (n = 12): group A (sepsis + EA at ST36), group B (sepsis + sham acupoint), group C (sepsis + vagotomy), and group D (sepsis + vagotomy + EA at ST36).

### 2.2. Experimental Methods

*(1) Model Making. *The cecal ligation and puncture (CLP) model was performed according to Chaudry's method [8]. The rats came to be operated after successful intramuscular anesthesia using Ketamine and Sumianxin (2:1 preparation, 0.4 ml/kg). During the operation, the rat's abdominal skin was prepared and cut open 2 centimeters along the midline. The cecum was carefully pulled out and punctured 3 times with NO.16 needle and its root ligated, which caused fistula intestine. To prevent the punctured pinhole from closing, one rubber stripe (2 cm× 2 mm) was fixed in it to drainage. The cecum was inserted back into the abdomen, which was closed then with NO. 0 silk. The rats were given subcutaneous injection of physiological saline (50 mL/kg) for antishock and had free access to water after surgery.

*(2) Processing Methods. *All rats in this experiment were performed sepsis by CLP. Animals of groups A, B, and D were fixed with a bag (limbs exposed through four holes) and acupunctured when they were awake about 40 minutes after surgery. Zusanli point (ST36) is outside of the knee, about 5 mm below the fibular head [3]. The nonacupoint locates below the outer of ST36 about 5mm. The two needles were connected with two electrode coils, and the acupoint was stimulated continuously (2 mA, 2-100Hz) for 1 hour with a special device (HANS, LH202H, Beijing, China). The nonacupoint was stimulated using the same electrode, parameters of intensity, frequency, and time (2 mA, 2-100Hz, 1h). In groups C and D, abdominal vagotomy [9] was operated before CLP.

The rat's abdominal skin was cut open 2 centimeters along the midline after successful intramuscular anesthesia. When the stomach was gently pulled down and twisted, the vagus nerve branches of the esophagus were exposed. After that, the branches were bluntly separated, which fixed and transected by a nylon cord. All rats had free access to water and were sacrificed at 6 hours after surgery.

### 2.3. Observations and Detection Methods

#### 2.3.1. Creatine Kinase-MB (CK-MB) Content

Six hours after CLP, all rats were hocussed with Ketamine and Sumianxin (2:1 preparation, 0.5 ml/kg); then abdominal arterial blood was extracted 2ml, and the CK-MB content of which was detected by automatic analyzer.

#### 2.3.2. Content of Tumor Necrosis Factor (TNF-*α*), Nitric Oxide (NO), and Myeloperoxidase (MPO)

Animals were sacrificed by draining abdominal aortic blood. A part of cardiac muscle was clipped and packed in liquid nitrogen cryopreservation. 100 mg liver and 9 ml saline were added to prepare a 10 % homogenate. Then it was centrifuged for 10–30 minutes with a speed of 1000 and 3000 rev/min, respectively (with the speed depending on the measurement targets). The supernatant after centrifuging was cryopreserved (-80°C) and prepared to be detected.

*(1) TNF-α Content. *A rat TNF-*α* ELISA test kit (Diaclone Company) was used (sensitivity 20 pg/ml, detection range 20-1000 pg/ml). In strict accordance with the manual steps, the instrument automatically calculated a standard curve regression equation: y = 0.0173 + 0.0003x, r = 0.9942. The optical density of the sample was put into a standard curve and multiplied by the appropriate dilution factor to calculate the sample's TNF-*α* content, with a unit of pg/ml.

*(2) NO and MPO Content. *An NO or MPO kit (Nanjing Jiancheng Bioengineering Institute) was used in strict accordance with the instructions. The result of NO was expressed in *μ*mol/gprot and MPO in U/gprot.

#### 2.3.3. Moisture Rate of Cardiac Tissue

After animals were sacrificed, a part of cardiac tissue was clipped, weighed, and then put into the oven (90°C) to bake for 72 hours. The drying tissue was also weighed.

### 2.4. Statistical Analysis

ANOVA statistical analysis software was used to process the data. Differences between groups were compared (indicated with $\overset{-}{x}\pm s$).* P* <0.05 was considered statistically significant. The original data can be found in the Department of Rehabilitation of the 309th Hospital of People's Liberation Army in Beijing.

## 3. Results

### 3.1. Plasma CK-MB Content

Six hours after CLP, the CK-MB content of all rats was increased in different degrees; that of group B decreased compared to the other groups (*P*<0.01). There was no significant difference between group C and group D (*P*>0.05) (see Table 1).

### 3.2. Content of TNF-*α*, NO, and MPO and Moisture Rate in Cardiac Tissue

At 6 hours after CLP, the content of TNF-*α*, NO, and MPO and moisture rate in cardiac tissue of all groups showed a significant increase; those of group A were significantly lower than those in the other groups (*P*<0.01 or* P*<0.05). The above observed index showed no significant difference between groups C and D (*P*>0.05) (see Tables 2, 3, 4, and 5).

## 4. Discussion

Sepsis, a systemic inflammatory response syndrome caused by infection, can lead to septic shock and multiple organ dysfunction syndrome (MODS). The heart is one of vulnerable organs in sepsis. Clinical studies confirmed that the organic heart injury had been induced in early onset of sepsis, accompanied by hypotension, cardiac failure, and arrhythmia [10].

TNF-*α* is the initial secretion proinflammatory cytokines when body suffers from adverse stimuli, which can damage organs directly or by other ways [11]. Some researchers found that the TNF-*α* level significantly increased in patients with sepsis caused by severe burns and was positively correlated with serum myocardial enzymes [12]. When sepsis or MODS occur, NO is out of control and plays an important role in systemic infection and excessive pathophysiology of inflammatory response. It can aggravate organ damage of sepsis. NO is the substrate for MPO. The removal of NO by MPO can lower NO bioavailability and inhibit nitric oxide synthase (NOS) activity. In the previous series of studies, we found that the content of TNF-*α*, NO, NOS, and other inflammatory factors of liver or intestine tissue was significantly increased in sepsis and abdominal adhesion rats [13–16]. These abnormal elevated inflammatory factors lead to organ dysfunction [17].

Zusanli (ST36) is the confluent acupoint of Zu Yang Ming meridians, which can promote gastrointestinal motility and improve the blood flow, immunity, and other effects. In addition, modern research results show that acupuncturing at Zusanli plays important anti-inflammatory roles, which can reduce inflammatory factor levels abnormally elevated in blood plasma or tissue of sepsis, abdominal adhesion, endotoxemia, and scald rats and protect organ function [6, 9, 18–20].

In this study, the authors observed that 6 hours after CLP, the heart of animals had adverse plasma myocardial enzyme level and ischemia-reperfusion injury. The CK-MB content, tissue moisture rate, and the levels of TNF-*α*, NO, and MPO were significantly increased. Compared to other groups, those of group A (EA at Zusanli) showed lower levels, which indicates that acupuncturing at Zusanli had a protective effect on the myocardial injury of sepsis by inhibiting elevated levels of inflammatory cytokines.

The cholinergic anti-inflammatory pathway is to regulate against systemic inflammation, which goes through cholinergic nerve and its transmitters [21]. Direct electrical stimulation of cholinergic nerves (efferent vagus nerve) can inhibit the synthesis of TNF-*α* [21]. Hu et al. observed the effect of e vagus nerve electrical stimulation on acute lung injury and cardiac inflammation in endotoxemia rats. The results showed that vagus nerve stimulation could significantly reduce TNF-*α* and MPO activity in cardiopulmonary tissues and pathological damage [22, 23]. In the abdominal adhesion experiment, the authors found after cutting off the vagus nerve that the level of proinflammatory factors was increased. Even with stimulation of acupuncturing Zusanli, it showed less decline [4, 5, 13, 19, 24]. The integrity of vagus nerve plays an important role in anti-inflammatory and organ protection.

In this study, the authors also found that when the bilateral ventral vagus nerve is cut off, acupuncturing at Zusanli had less efficiency in reducing the inflammation level of sepsis rats and even more aggravation. The level of proinflammatory factors is balanced by sympathetic adrenergic nerve and parasymptomatic cholinergic nerve. When the parasymptomatic cholinergic nerve is blocked, the effect of sympathetic adrenergic nerve can be activated, leading to elevated level of proinflammatory cytokine.

## 5. Conclusion

EA at Zusanli had a positive role in myocardial injury of sepsis rats by reducing the high TNF-*α*, MPO, and NO content, lowering plasma activity of CK-MB and moisture content. The authors suggest that the anti-inflammatory and myocardial protective effects of EA at Zusanli in sepsis be related to the activation of cholinergic anti-inflammatory pathway.

## Figures and Tables

**Table 1:** Comparison of plasma CK-MB at 6 hours after CLP [($\overset{-}{x}\pm s$), U/L].

Groups	Rats	CK-MB	Compared with group A	Compared with group C
			*P*-value	*P*-value
A	12	3020±190	-	0.0001
B	12	3850±153	0.0001	0.0057
C	12	4106±246	0.0001	-
D	12	4090±257	0.0001	0.8776

**Table 2:** Comparison of TNF-*α* content in cardiac tissue at 6 hours after CLP [($\overset{-}{x}\pm s$), pg/ml].

Groups	Rats	TNF-*α*	Compared with group A	Compared with group C
			*P*-value	*P*-value
A	12	284.22±97.87	-	0.0001
B	12	444.23±111.20	0.0011	0.0136
C	12	572.45±122.80	0.0001	-
D	12	570.68±120.57	0.0001	0.9719

**Table 3:** Comparison of NO content in cardiac tissue at 6 hours after CLP [($\overset{-}{x}\pm s$), pg/ml].

Groups	Rats	NO	Compared with group A	Compared with group C
			*P*-value	*P*-value
A	12	2.09±0.20	-	0.0001
B	12	2.50±0.16	0.0003	0.0038
C	12	2.92±0.42	0.0001	-
D	12	2.84±0.25	0.0001	0.5765

**Table 4:** Comparison of MPO content in cardiac tissue at 6 hours after CLP [($\overset{-}{x}\pm s$), U/gprot].

Groups	Rats	MPO	Compared with group A	Compared with group C
			*P*-value	*P*-value
A	12	1.56±0.18	-	0.0001
B	12	1.80±0.24	0.0111	0.0004
C	12	2.19±0.22	0.0001	-
D	12	2.07±0.16	0.0001	0.1407

**Table 5:** Comparison of moisture rate in cardiac tissue at 6 hours after CLP [($\overset{-}{x}\pm s$), %].

Groups	Rats	Moisture rate	Compared with group A	Compared with group C
			*P*-value	*P*-value
A	12	66.2±1.9	-	0.0001
B	12	71.6±3.6	0.0001	0.0164
C	12	76.1±4.8	0.0001	-
D	12	76.4±4.4	0.0001	0.8747

## Data Availability

The data used to support the findings of this study are available from the corresponding author upon request.

## References

[1] Gao G., Feng Z., Chang Z. G., Tang P. X., Tong H. F. (2013). International guidelines on severe sepsis and septic shock treatment 2012. *Chinese Critical Care Medicine*.

[2] Xia J. D., Su Z., Zheng H. Y., Hua L. W. (2017). Prognostic value of a change in troponin-I? levels in patients with sepsis-associated myocardial dysfunction. *Journal of China Medical University*.

[3] Li Z. R. (2003). *Experimental Acupuncture*.

[4] Zhang L. J., Huang Z. J., Bai H. Y., Hu S., Shi X. (2011). The empirical study of electro-acupuncture at Zusanli points on abdominal adhesions. *Journal of Chinese Medicine*.

[5] Zhang L. J., Duan W. H., Huang Z. J., Hu S., Shi X. (2014). Effect of Electro-acupuncture at Zusanli point on expression of VEGF in postoperative intra-abdominal adhesions. *China Journal of Chinese Medicine*.

[6] Shi X., Song Q., Hu S., Li Z., Liu Q., Wang L. (2008). Study on protective action of electroacupuncture on endotoxin-induced hepatic injury in rats. *Chinese Acupuncture & Moxibustion*.

[7] Hu S., Song Q., Wang H. B. (2007). Study on the protective effect and mechanism of electro-acupuncturing at Zusanli point on endotoxin induced hepatic injury in rats. *Chinese Journal of Integrated Traditional and Western Medicine in intensive and Critical Care*.

[8] Chaudry I. H., Wichterman K. A., Baue A. E. (1979). Effect of sepsis on tissue adenine nucleotide levels. *Surgery*.

[9] Hu S., Song Q., Wang L., Lv Y., Zhou G. Y., Sheng Z. Y. (2008). Effect of activating cholinergic anti-inflammatory pathway by electro-acupuncture on proinflammatory cytokines release and organ dysfunction in rat with endotoxin challenge. *Chinese Journal of Integrated Traditional and Western Medicine in intensive and Critical Care*.

[10] Turner A., Tsamitros M., Bellomo R. (1999). Myocardial cell injury in septic shock. *Critical Care Medicine*.

[11] Wang H., Yu M., Ochani M. (2003). Nicotinic acetylcholine receptor *α*7 subunit is an essential regulator of inflammation. *Nature*.

[12] Wan F. S. (2004). Progress in myocardial damage after severe burns. *Acute Academic Medicine*.

[13] Zhang L. J., Wang H. Z., Huang Z. J., Hu S., Shi X. (2016). Inhibitory effect of electro-acupuncture at Zusanli point on flammatory factors of postoperative intra-abdominal adhesions. *Military Medicine Science*.

[14] Hu S., Zhang L., Bai H., Bao C. (2009). The effects of electro-acupuncturing at Zusanli point on intestinal proinflammatory factors, diamine oxidase and tissue water content in rats with sepsis. *Chinese Critical Care Medicine*.

[15] Hu S., Zhang L. J., Bai H. Y., Bao C. M. (2010). Effects of electro-acupuncture at Zusanli point on the expression of proinflammatory cytokines, the activity of diamine oxidase and the rate of water content in the small intestine in rats with sepsis. *World Chinese Journal of Digestology*.

[16] Hu S., Zhang L. J., Bai H. Y., Bao C. M. (2009). The protective effect of electro-Acunpunture at Zusanli Point on proinflammatory factors induced-hepatic in rats with sepsis. *Journal of Medicine Research*.

[17] Hu S., Zhang L. J., Bai H. Y., Tian Y. J. (2010). Effect of electro-acupuncturing at Zusanli point on tumor necrosis factor-аinduced multiple organ dysfunction. *Chinese Journal of Pathophysiology*.

[18] Hu S., Wang L., Song Q. (2009). Effect and mechanism of electro-acupuncture at Zusanli on gastrointestinal mucosal blood flow and motility in rats with scald injury. *Chinese Journal of Integrated Traditional and Western Medicine in intensive and Critical Care*.

[19] Zhang L. J., Huang Z. J., Hu S., Shi X. (2012). The empirical study of electro-acupuncture at Zusanli points on angiogenesis of intra-abdominal adhesion tissues. *Journal of Chinese Medicine*.

[20] Shi X., Zhang L. J., Bai H. Y., Bao C. M., Hu S., Guan L. (2010). Effects of electro-acupuncture on hepatic blood flow and lipid peroxidation in septic rats. *Chinese Acupuncture And Moxibustion*.

[21] Borovikova L. V., Ivanova S., Zhang M. (2000). Vagus nerve stimulation attenuates the systemic inflammatory response to endotoxin. *Nature*.

[22] Shi D. G., Hu S., Jiang X. G. (2003). Effects of efferent vagus nerve excitation on inflammatory in heart tissue in rats with endotoxemia. *Chinese Critical Care Medicine*.

[23] Shi D. G., Hu S., Jiang X. G. (2002). Effects of efferent vagus nerve excitation on acute lung injury induced by endotoxemia. *Chinese Critical Care Medicine*.

[24] Zhang L., Wang H., Huang Z. (2014). Inhibiting effect of electroacupuncture at Zusanli on early inflammatory factor levels formed by postoperative abdominal adhesions. *Evidence-Based Complementary and Alternative Medicine*.

